# A new species of *Brachycephalus* (Anura: Brachycephalidae) from Santa Catarina, southern Brazil

**DOI:** 10.7717/peerj.2629

**Published:** 2016-10-27

**Authors:** Marcos R. Bornschein, Luiz F. Ribeiro, David C. Blackburn, Edward L. Stanley, Marcio R. Pie

**Affiliations:** 1 Campus do Litoral Paulista, Universidade Estadual Paulista, São Vicente, São Paulo, Brazil; 2 Mater Natura - Instituto de Estudos Ambientais, Curitiba, Paraná, Brazil; 3 Escola de Ciências da Vida, Pontifícia Universidade Católica do Paraná, Curitiba, Paraná, Brazil; 4 University of Florida, Florida Museum of Natural History, Gainesville, FL, USA; 5 Departamento de Zoologia, Universidade Federal do Paraná, Brazil

**Keywords:** Abundance, Conservation, Microendemism, Atlantic Rainforest, Montane forest, Microcomputed tomography, Serra do Mar

## Abstract

A new species of *Brachycephalus* (Anura: Brachycephalidae) is described from the Atlantic Forest of northeastern state of Santa Catarina, southern Brazil. Nine specimens (eight adults and a juvenile) were collected from the leaf litter of montane forests 790–835 m above sea level (a.s.l.). The new species is a member of the *pernix* group by its bufoniform shape and the absence of dermal co-ossification and is distinguished from all its congeners by a combination of its general coloration (dorsal region of head, dorsum, legs, arms, and flanks light, brownish green to dark, olive green, with darker region in the middle of the dorsum and a white line along the vertebral column in most specimens) and by its smooth dorsum. The geographical distribution of the new species is highly reduced (extent of occurrence estimated as 25.04 ha, or possibly 34.37 ha). In addition, its habitat has experienced some level of degradation, raising concerns about the future conservation of the species. Preliminary density estimates suggest one calling individual every 3–4 m^2^ at 815–835 m a.s.l. and every 100 m^2^ at 790 m a.s.l. Together with the recently described *B. boticario* and *B. fuscolineatus*, the new species is among the southernmost species of *Brachycephalus* known to date.

## Introduction

*Brachycephalus* Fitzinger, 1826 is a remarkable genus of miniaturized frogs endemic to the Brazilian Atlantic Forest (Pie et al., 2013; Bornschein et al., 2016). *Brachycephalus* species often display an extraordinarily high level of endemism, with one or a few adjacent mountaintops representing the entire known geographical ranges of many species (e.g., Ribeiro et al., 2015; Bornschein et al., 2016). Such extreme endemism is probably due to their particular cold/humid environmental requirements found in high-elevation regions, as in the case of cloud forests (see Pie et al., 2013; Bornschein et al., 2016). Firkowski et al. (2016) recently studied the process of diversification of montane *Brachycephalus* species using a combination of species delimitation methods and molecular divergence dating. Their study suggests a scenario of isolation in “sky islands” through a combination of niche conservatism and climatic changes in the Brazilian Atlantic Forest during the Quaternary. Based on this scenario, one could expect that *Brachycephalus* is likely to be found in montane regions of the Brazilian Atlantic Forest with similar climatic conditions, including the possibility of new species. Indeed, over the last decade, 16 new *Brachycephalus* species were described (Alves et al., 2009; Haddad et al., 2010; Pombal Jr & Izecksohn, 2011; Napoli et al., 2011; Clemente-Carvalho et al., 2012; Garey et al., 2012; Condez et al., 2014; Condez et al., 2016; Pie & Ribeiro, 2015; Ribeiro et al., 2015), suggesting that species diversity of the genus may still be underestimated.

The species of *Brachycephalus* have been recently divided into three species groups (Ribeiro et al., 2015): *ephippium* (Spix, 1824), *didactylus* (Izecksohn, 1971), and *pernix* Pombal Jr, Wistuba & Bornschein, 1998. The latter included the species of *Brachycephalus* that shared their bufoniform body shape with the *ephippium* group, but lacked their characteristic dermal ossification, namely *B. pernix* Pombal Jr, Wistuba & Bornschein, 1998, *B. brunneus* Ribeiro, Alves, Haddad & Reis, 2005, *B. izecksohni* Ribeiro, Alves, Haddad & Reis, 2005, *B. ferruginus* Alves, Ribeiro, Haddad & Reis, 2006, *B. pombali* Alves, Ribeiro, Haddad & Reis, 2006, *B. tridactylus* Garey, Lima, Hartmann & Haddad, 2012, *B. auroguttatus* Ribeiro, Firkowski, Bornschein & Pie, 2015, *B. boticario* Pie, Bornschein, Firkowski, Belmonte-Lopes & Ribeiro, 2015, *B. fuscolineatus* Pie, Bornschein, Firkowski, Belmonte-Lopes & Ribeiro, 2015, *B. leopardus* Ribeiro, Firkowski & Pie, 2015, *B. mariaeterezae* Bornschein, Morato, Firkowski, Ribeiro & Pie, 2015, *B. olivaceus* Bornschein, Morato, Firkowski, Ribeiro & Pie, 2015, *B. quiririensis* Pie & Ribeiro, 2015, and *B. verrucosus* Ribeiro, Firkowski, Bornschein & Pie, 2015 (Pie & Ribeiro, 2015; Ribeiro et al., 2015), for a total of 14 described species. In the present study, we describe a new species of the *pernix* species group from northeastern state of Santa Catarina, southern Brazil. This discovery is part of a continuing effort to investigate montane anurans of southern Brazil (see Bornschein et al., 2015; Bornschein et al., 2016; Pie & Ribeiro, 2015; Ribeiro et al., 2015; Firkowski et al., 2016).

## Materials and Methods

Specimens were collected according to permits issued by ICMBIO–SISBIO (no. 10.500, 22470–2/1911426) and are deposited in the Museu de História Natural Capão da Imbuia (MHNCI), Curitiba, state of Paraná, Brazil and in the Museu Nacional (MNRJ), Rio de Janeiro, state of Rio de Janeiro, Brazil. In addition, we examined specimens deposited in the following (Brazilian) collections: Célio F. B. Haddad collection (CFBH), Departamento de Zoologia, Universidade Estadual Paulista, Campus de Rio Claro, state of São Paulo; MHNCI; Coleção Herpetológica do Departamento de Zoologia (DZUP), Universidade Federal do Paraná, Curitiba, state of Paraná; MNRJ; Museu de Zoologia da Universidade de São Paulo (MZUSP), São Paulo, state of São Paulo; and Museu de História Natural (ZUEC), Universidade Estadual de Campinas, Campinas, state of São Paulo. A list of the examined specimens is provided in Appendix S1.

Measurements were made with a micrometric eyepiece attached to a stereomicroscope. Measurements abbreviations were as follows (Lynch & Duellman, 1997; Heyer et al., 1990): snout-vent length (SVL); head length, from tip of snout to angle of jaw (HL); head width—greatest width of head located between angles of jaw (HW); eye diameter (ED); nostril diameter (ND); interorbital distance, between anterior corners of the eyes (IOD); internostril distance, between inner margins of nostrils (IND); eye-nostril distance, from anterior corner of the eye to posterior margin of nostril (END); thigh length (THL); and tibia length (TBL). The classification of Brazilian vegetation proposed by the RADAMBRASIL project (*in* Veloso, Rangel-Filho & Lima, 1991) was used to characterize the habitat in the type locality. Altitudinal records were obtained after plotting the geographical coordinates of the lowest and highest altitudinal records in the field using Google Earth. We obtained preliminary data on individual density by slowly walking across the study area for 2 h and placing markings on the vegetation whenever an individual was heard. We then measured the extent of the sampling area (= the extent of the auditory sampling) and counted the number of markings per sampled area. We recognize that this is an imprecise estimate, but given the scarcity of data on basic natural history of most *Brachycephalus* species, we believe that this information is still valuable.

High resolution micro-computed tomography (Micro-CT) scans were produced for one paratype at the University of Florida’s Nanoscale Research Facility, using a Phoenix v|tome|x M (GE’s Measurement & Control business, Boston, USA) with a 180 kv x-ray tube and a diamond-tungsten target, with the following settings: 60 kV, 175 mA, a one second detector time, averaging of three images per rotation and a voxel resolution of 10.52 µm. Raw x-ray data were processed using GE’s proprietary datos|x software v 2.3 to produce a series of tomogram images. These Micro-CT image stacks were then viewed, sectioned, measured, and analyzed using VG StudioMax 3.0 (Volume Graphics, Heidelberg, Germany). Final figures were prepared with Photoshop and Illustrator (CS5, Adobe). Tomograms (TIF format) and shape files (STL format) are freely available from http://morphosource.org/Detail/MediaDetail/Show/media_id/10212.

The electronic version of this article in Portable Document Format (PDF) will represent a published work according to the International Commission on Zoological Nomenclature (ICZN), and hence the new name contained in the electronic version is effectively published under that Code from the electronic edition alone. This published work and the nomenclatural act it contains has been registered in ZooBank, the online registration system for the ICZN. The ZooBank LSIDs (Life Science Identifiers) can be resolved and the associated information viewed through any standard web browser by appending the LSID to the prefix “http://zoobank.org/”. The LSID for this publication is: 32F6D8BC-7B15-4A81-BC82-D4DEE1B38DD8. The online version of this work is archived and available from the following digital repositories: PeerJ, PubMed Central, and CLOCKSS. It is important to note that the erection of this new species is a hypothesis based on currently available evidence. Further evaluations, improved for example by bioacoustics and molecular methods, will be valuable to corroborate its distinctiveness.

## Results

*Brachycephalus* ***albolineatus*** sp. nov.

**Figure 1:**
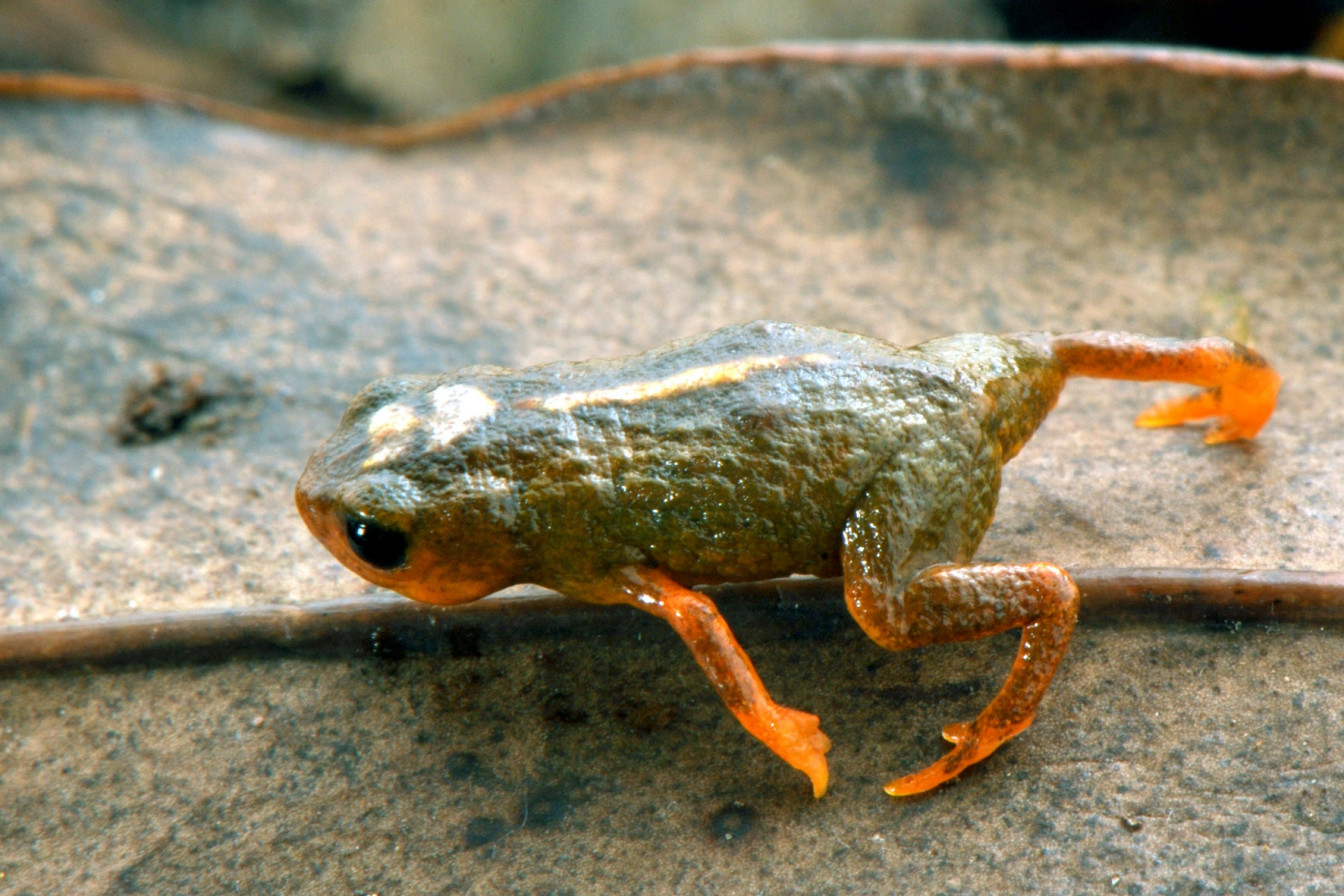
Holotype of *Brachycephalus albolineatus* in life.

**Figure 2:**
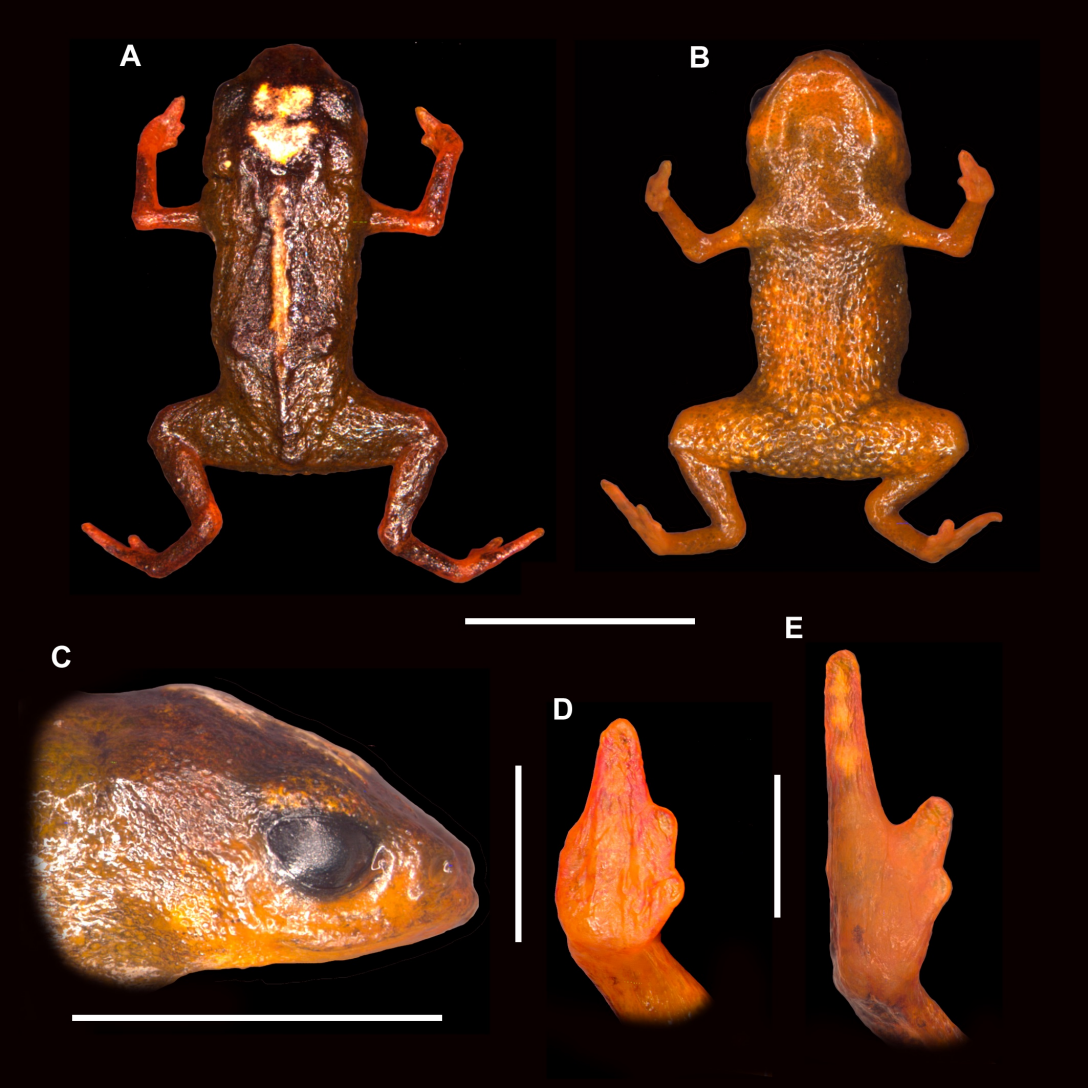
Holotype of *Brachycephalus albolineatus* (MHNCI 10290). The image was obtained a few minutes after being sacrificed and fixed in formalin. (A) Dorsal view of the body; (B) ventral view of the body; (C) lateral view of the head; (D) ventral view of right hand; and (E) ventral view of right foot. Vertical scale bars = 1 mm. Horizontal scale bars = 5 mm.

**Figure 3:**
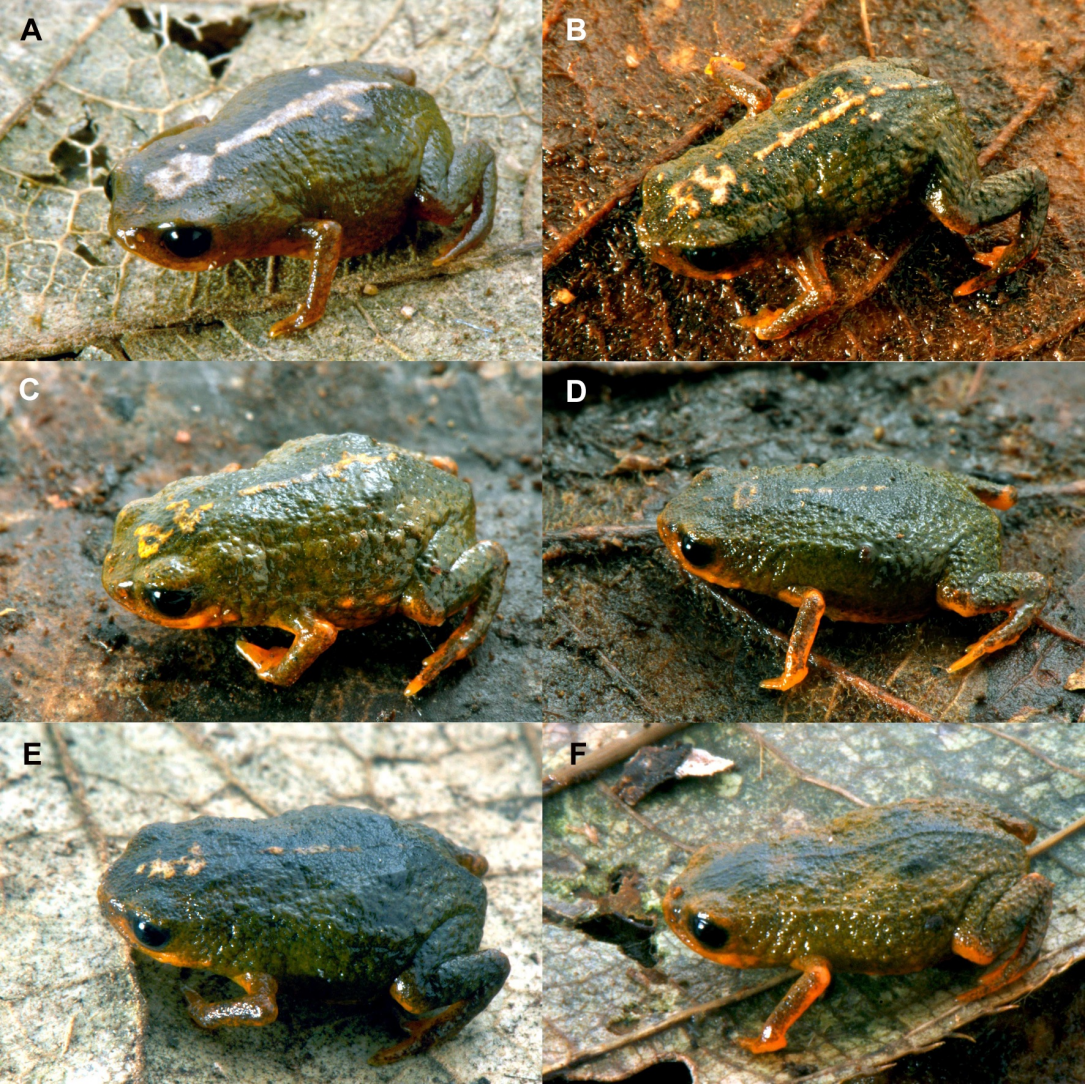
Variation in dorsal coloration among paratypes of *Brachycephalus albolineatus*. (A), MHNCI 10295; (B), MHNCI 10299; (C), MHNCI 10297; (D), MHNCI 10298; (E), MHNCI 10300; (F), MHNCI 10296. Notice a line of protruding glands on the dorsolateral region of body (although the degree of conspicuousness of this line varied among individuals). Also, the white line on the middle of the dorsum is completely absent in specimen “F”.

**Figure 4:**
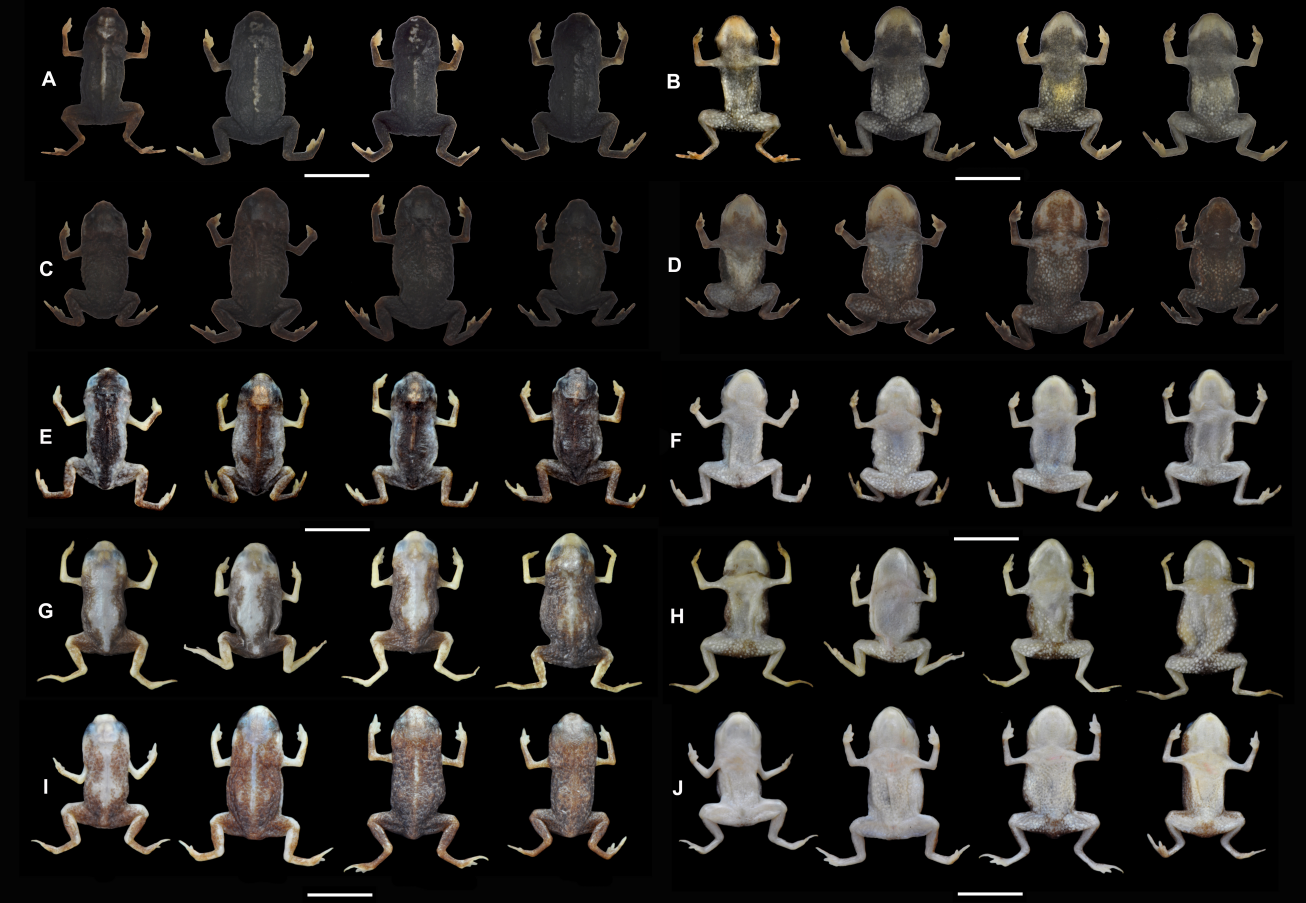
Comparison between *B. albolineatus* and other species of *Brachycephalus* that are geographically close, in preservative. (A) dorsal variation in *B. albolineatus;*(B) ventral variation in *B. albolineatus* (from left MHNCI 10290, 10297, 10295, 10296); (C) dorsal variation in *B. olivaceus*; (D) ventral variation in *B. olivaceus* (MHNCI 9815, 9814, 9813, 9816); (E) dorsal variation in *B. fuscolineatus*; (F) ventral variation in *B. fuscolineatus* (MHNCI 10236, 10231, 10235, 10237); (G) dorsal variation in *B. boticario*; (H) ventral variation in *B. boticario* (from the left MHNCI 10731, 10253, 10257, 10255); (I) dorsal variation in *B. verrucosus*; (J) ventral variation in *B. verrucosus* (MHNCI 10223, 10219, 10226, 10225). Dorsal and ventral views are shown with specimens in the same order. We selected the specimens with the most variation in color to illustrate intraspecific variation. Scale bars = 5 mm.

**Figure 5:**
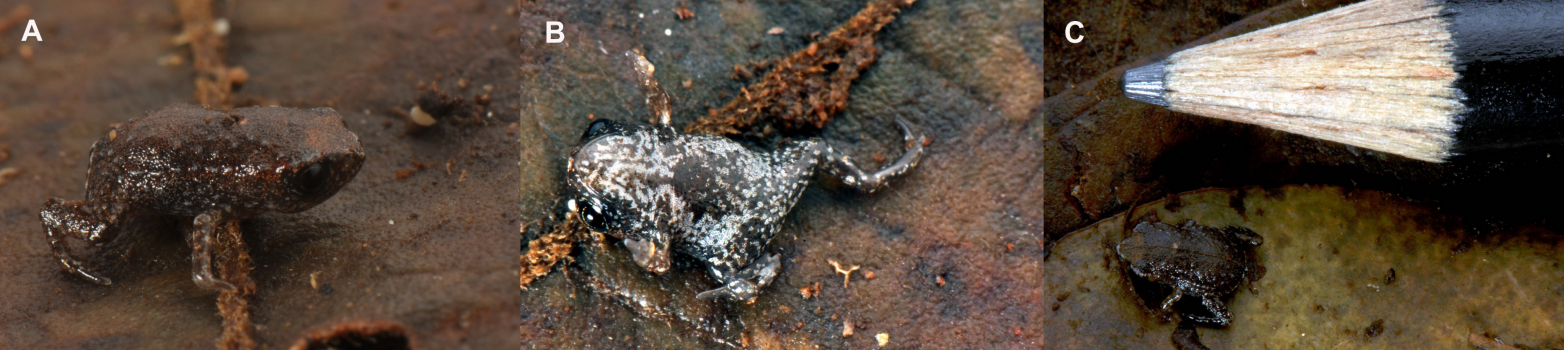
Juvenile of *Brachycephalus albolineatus* (MHNCI 10293). (A) dorsal view; (B) ventral view; (C) general view next to the tip of a pencil, for scale.

**Figure 6:**
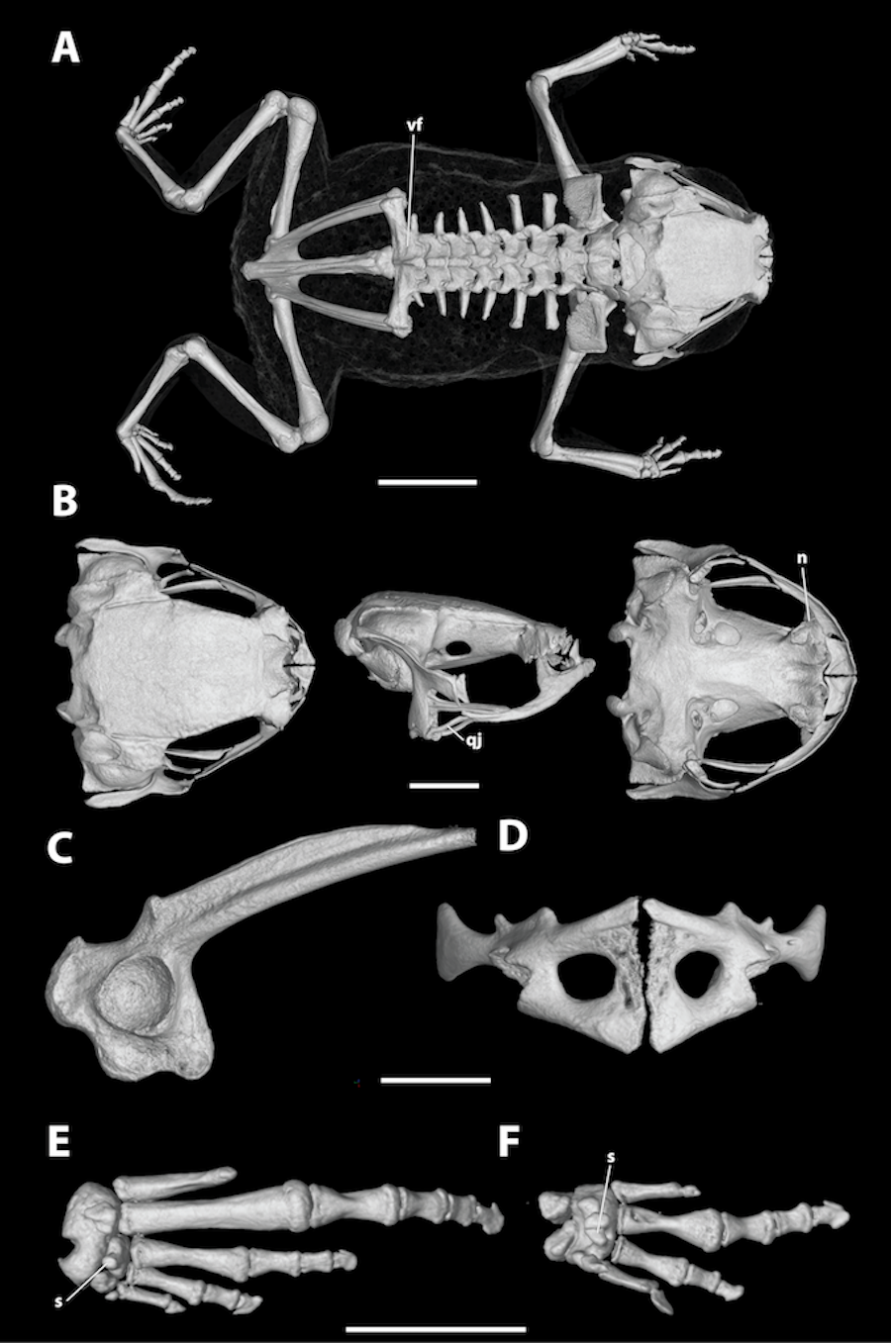
High-resolution computed tomography (Micro-CT) scans of a paratype of *Brachycephalus albolineatus* (MHNCI 10295) showing key osteological features. (A) Dorsal view of the skeleton; (B) dorsal, lateral, and ventral views of the skull (without the lower jaw); (C) ilium in lateral view; (D) pectoral girdle in ventral view; (E) right foot in plantar view; and (F) right hand in palmar view. Abbreviations: n, neopalatine; qj, quadrojugal; s, sesamoid; vf, vertebral fusion between presacral vertebra VIII and sacral vertebra. Scale bars equal 2 mm.

Urn:lsid:zoobank.org:act: 250B70C4-8FB0-44B6-9D05-CC2955E0748F (Figs.1–6)

**Holotype.** MHNCI 10290 (Figs. 1 and 2) adult male, collected at Morro Boa Vista (26°30′58″S, 49°03′14″W; 835 m a.s.l.), on the border between the municipalities of Jaraguá do Sul, and Massaranduba, state of Santa Catarina, southern Brazil, on 25 October 2012 by MRP, LFR, and Felipe A. Cini da Silva.

**Paratopotypes.** MHNCI 10295–10300, MNRJ 90349 (Figs. 3–5), all adult males, collected on 5–6 February 2016 by MRB & LFR.

**Referred specimens.** MHNCI 10293, juvenile (Fig. 6), collected together with some of the paratopotypes on 5 February 2016 by MRB & LFR.

**Diagnosis.** *Brachycephalus albolineatus* is a member of the genus *Brachycephalus* based on diagnostic morphological traits, including phalangeal reduction, an arciferal pectoral girdle in which the ossified procoracoid and epicoracoid cartilages are fused to the clavicle, coracoid, and scapula, a suprascapula expanded with a prominent cleithrum, and the absence of a sternum (modified from Kaplan (2002), Izecksohn (1971), Ford & Cannatella (1993), Ribeiro et al. (2005), Alves et al. (2006) and Da Silva, Campos & Sebben (2007); Fig. 5). *Brachycephalus albolineatus* is a member of the *pernix* group, as defined by Ribeiro et al. (2015), by having a bufoniform body and lacking dermal co-ossification. Within *Brachycephalus*, *B. albolineatus* is distinguished from all of the species in the genus by the following combination of characters: (1) body bufoniform; (2) absence of dermal co-ossification; (3) adult size SVL 9.9–11.4 mm; (4) dorsum smooth (Fig. 1); (5) fusion of the last presacral (VIII) and sacral vertebrae; (6) general color (in life) of the dorsal region of head, dorsum, legs, arms and flanks light, brownish green to dark, olive green, always with a dark green region along the middle of the dorsum and a white line along the vertebral column in most specimens.

**Comparison with other species.** *Brachycephalus albolineatus* is unique among other species of its genus by its distinctive white thin stripe with minute yellow dots along its vertebral column, present in most specimens. *Brachycephalus boticario*, *B. mariaeterezae*, *B. quiririensis*, *B. guarani* Clemente-Carvalho, Giaretta, Condez, Haddad & Reis, 2012, some *B. ferruginus*, and some *B. verrucosus* also have mid-dorsal stripes but with different coloration: yellow in *B. boticario*, * B. quiririensis*, *B. auroguttatus*, and *B. verrucosus*, light blue in *B. mariaeterezae*, brown in *B. guarani* (Clemente-Carvalho et al., 2012), and reddish-brown in *B. ferruginus*. The dorsum of these species is also different: dark green in the middle of the dorsum and green in the remaining of the dorsum in the new species, as opposed to yellow in *B. mariaeterezae*, brown in *B. quiririensis*, orange in *B. ferruginus* and *B. guarani* (Clemente-Carvalho et al., 2012), light brown in *B. boticario*, light green in *B. verrucosus*, brown in *B. quiririensis*, and pure yellow anteriorly and increasingly mixed with brown instead of yellow toward the posterior region in *B. auroguttatus*. *Brachycephalus albolineatus* is also distinguished from all these species by its coloration on ventral surface, being orange with brownish-green regions instead of orange in *B. boticario* and *B. guarani* (Clemente-Carvalho et al., 2012), yellow in *B. verrucosus*, yellow with small brown spots in *B. mariaeterezae*, orange with small green spots in *B. ferruginus*, orange anteriorly and brown with orange spots posteriorly in *B. quiririensis*, and orange mixed with brown in *B. auroguttatus*. The *B. albolineatus* specimen lacking the white line (Fig. 3F) resembles *B. olivaceus*, but this species is uniformly dark-green or dark brown on the dorsum instead of a deep dark green on the center of the dorsum and green on the remaining dorsum in *B. albolineatus*. The *B. albolineatus* specimen that lacks the white line along the vertebral column (Fig. 3F) is also reminiscent of some specimens of *B. fuscolineatus*, but nevertheless can be safely distinguished from that species by green flanks (in contrast with the yellow flanks of *B. fuscolineatus*), deep dark-green on the middle of the dorsum (instead of dark-brown to black of *B. fuscolineatus*), and an orange and brownish-green venter (instead of a uniformly orange venter of *B. fuscolineatus*). The new species is also distinguished from *B. mariaeterezae*, *B. olivaceus*, *B. auroguttatus*, *B. verrucosus*, *B. fuscolineatus*, *B. leopardus*, *B. boticario*, and *B. quiririensis* by having a smooth dorsum, as opposed to their rough dorsum. In preservative, we highlighted the distinctiveness of *B. albolineatus* in relation to geographically close species in Fig. 4. The only species that resembles *B. albolineatus* in preservative is *B. olivaceus* (Figs. 4C and 4D), which is only clearly distinguishable in specimens with the white dorsal stripe. *Brachycephalus albolineatus* is easily distinguished from the remaining species of the *pernix* group by the general green coloration on dorsum, instead of (1) dark brown in *B. brunneus*, (2) orange (or orange and yellow), normally with dark spots on the flanks, of *B. izecksohni*, *B. leopardus*, *B. pombali*, and *B. tridactylus*, and (3) orange on head and on central dorsum with black remaining parts of *B. pernix*. The bufoniform body shape, white stripe along the vertebral column, and the two shades of green on the dorsum also distinguish the new species from all species of the *didactylus* group, namely *B. didactylus* (Izecksohn, 1971), *B. hermogenesi* (Giaretta & Sawaya, 1998), *B. pulex* Napoli, Caramaschi, Cruz & Dias, 2011, and *B. sulfuratus* Condez, Monteiro, Comitti, Garcia, Amaral & Haddad, 2016, accordingly Ribeiro et al. (2015) and this study, which have leptodactyliform body shapes and homogeneous dorsal coloration, at times with an “X”-shaped darker mark on their dorsum (Izecksohn, 1971; Giaretta & Sawaya, 1998; Napoli et al., 2011; Condez et al., 2016). *Brachycephalus albolineatus* is distinguished from all species of the *ephippium* group whose members show dermal co-ossification, accordingly Ribeiro et al. (2015), which is absent in the new species. Apart from the difference in coloration between the new species and all species of the *ephippium* group, it also differs in body size, which is larger in adults of the *ephippium* group, such as *B. alipioi* Pombal Jr & Gasparini, 2006 (SVL = 12.5–16.2 mm; Pombal Jr & Gasparini (2006)), *B. crispus* Condez, Clemente-Carvalho, Haddad & Reis, 2014 (SVL = 11.5–15.6 mm; Condez et al. (2014)), *B. margaritatus* Pombal Jr & Izecksohn, 2011 (SVL = 15.0–18.9 mm; Pombal Jr & Izecksohn (2011)), *B. pitanga* Alves, Sawaya, Reis & Haddad, 2009 (SVL = 10.8–12.1 mm; Alves et al. (2009)), *B. toby* Haddad, Alves, Clemente-Carvalho & Reis, 2010 (SVL = 11.3–14.2 mm; Haddad et al. (2010)), *B. vertebralis* Pombal Jr, 2001 (SVL = 10.5–14.2 mm; Pombal Jr (2001)), *B. nodoterga* Miranda-Ribeiro, 1920, *B. garbeanus* Miranda-Ribeiro, 1920, and *B. bufonoides* Miranda-Ribeiro, 1920 (SVL = 12.4 mm, 17.6 mm, and 13.5 mm, respectively; Pombal Jr (2010)).

**Description of the Holotype.** Body robust, bufoniform; head slightly wider than long (HW 120% of HL); HL 30% of snout-vent length; snout short, with length almost equal to ED, rounded in dorsal and lateral views nostrils protuberant, directed anterolaterally; canthus rostralis not distinct; lips nearly sigmoid; loreal region slightly concave; ED 39% of head length; tympanum indistinct; vocal sac not expanded externally; vocal slits present; tongue longer than wide, with posterior half not adherent to floor of mouth; choanae relatively small and round; vomerine odonthophores absent. Arm and forearm slender; arm approximately as long as forearm; tip of finger I and II slightly rounded, tip of finger III pointed; relative lengths of fingers IV < I < II < III ; subarticular tubercles absent; inner and outer metacarpal tubercles absent; legs short, moderately robust; thigh length 36% of snout-vent length, tibia length 84% of thigh length; toes II–IV short, relatively distinct; toe I reduced, toe V externally absent; relative length of toes II < III < IV, subarticular tubercles and inner metatarsal tubercles absent; outer metatarsal tubercle distinct, large, ovoid; dorsum smooth; head and arms smooth; dorsal region of the legs granular, glands circular, not juxtaposed; sides of the body granular, with glands circular, almost juxtaposed; belly granular, with glands circular; chin smooth.

**Coloration of the Holotype.** In life, the dorsum, head, sides of the body, dorsal region of arms, and legs are green, but becoming dark green in the center of the dorsum. The irregular dorsal line extending from the top of the head and along the vertebral column is white with minute yellow dots. The belly, chin, and ventral regions of arms and legs are orange with brownish green regions near the chest, inguinal region and cloaca. The iris is black (Figs. 1 and 2). In preservative, the orange coloration becomes pale cream, while brownish green coloration becomes darker (Figs. 4A and 4B).

**Measurements of Holotype.** SVL = 9.9, HL = 3.0, HW = 3.6, ED = 1.2, ND = 0.2, IOD = 2.0, THL = 3.6, TBL = 3.3, IND = 1.2, END = 0.5 (all measurements in mm).

**Variation in the Type Series.** Measurements and proportions of eight adults in millimeters are (mean ± SD, with range in parentheses): SVL 10.74 ± 0.52 (9.9–11.4); HL 3.34 ± 0.20 (3.0–3.6); HW 4.04 ± 0.28 (3.6–4.4); ED 1.26 ± 0.07 (1.2–1.4); ND 0.18 ± 0.03 (0.15–0.20); IOD 2.11 ± 0.10 (2.0–2.2); IND 1.21 ± 0.03 (1.2–1.3); END 0.52 ± 0.04 (0.5–0.6); THL 3.70 ± 0.12 (3.6–3.9); TBL 3.32 ± 0.17 (3.0–3.6). There is variation in the hue of the dorsal green coloration, from lighter, brownish green to darker, olive green. Also, the dark green region in the middle of the dorsum and the breadth of the white line along the vertebral column vary in breadth, with the white line being absent in one specimen (Fig. 3F). All paratypes have a line of protruding glands on the dorsolateral region of body, although the degree of conspicuousness of this line varies among individuals (Figs. 3A–3F). Preserved specimens become dark brown, while maintaining the distinct white stripe along their dorsum, but with varying widths among specimens (Fig. 4A). Ventral coloration tends to be dark brown, with pale cream regions of varying extents in the throat, ventral region of the thighs and belly, with some individuals still showing vestiges of yellow in their belly (Fig. 4B).

**Description of general osteology.** Based on Micro-CT scan of MHNCI 10295 (male; Fig. 5). The skull of *B. albolineatus* is characterized by having a robust neurocranium, lacking teeth, having a robust operculum covering the fenestra ovalis, lacking hyperossification, and having a slender quadratojugal, a relatively robust pterygoid, and a ‘complete’ squamosal with a prominent zygomatic ramus. The vomers are co-ossified to the sphenethmoid. The sphenethmoid appears co-ossified to the parasphenoid and the neopalatine is present. There are prominent ossified posteromedial processes from the hyoid. The arytenoid cartilages are heavily mineralized. The epicoracoid and procoracoid cartilages are ossified and fused to the clavicle, coracoid, and scapula to form a robust arciferal pectoral girdle. A bony sternum is absent. The last presacral vertebra (VIII) is fused to the sacral vertebra. The phalangeal formula for the manus is 1-2-3-1 and there is both a single ossified prepollex and a small palmar sesamoid. The phalangeal formula for the pes is 1-2-3-4-0 and, similar to the manus, there is a single ossified prehallux and a plantar sesamoid.

**Description of the juvenile.** The juvenile of *B. albolineatus* (MHNCI 10293, SVL = 5 mm) was found underneath a layer of leaf litter in the same location as the adults (Fig. 6). Its coloration is markedly different that of the adults, being dark brown, with light gray dots on the side of the body and on the dorsal region of the arms and legs. A straight line of light gray dots extends slightly above the direction of the eye to the posterior portion of the body. On the ventral surface, including the ventral side of arms and legs, the juvenile is black with white dots or with white lines. The skin on the dorsum is smooth, and the iris is black surrounded by a golden ring (Fig. 6).

**Etymology**. The specific epithet is from the Latin *albus* (“white”) and *lineatus* (“of a line”), in reference to the characteristic white stripe across the dorsum of the new species, present in most specimens.

**Figure 7:**
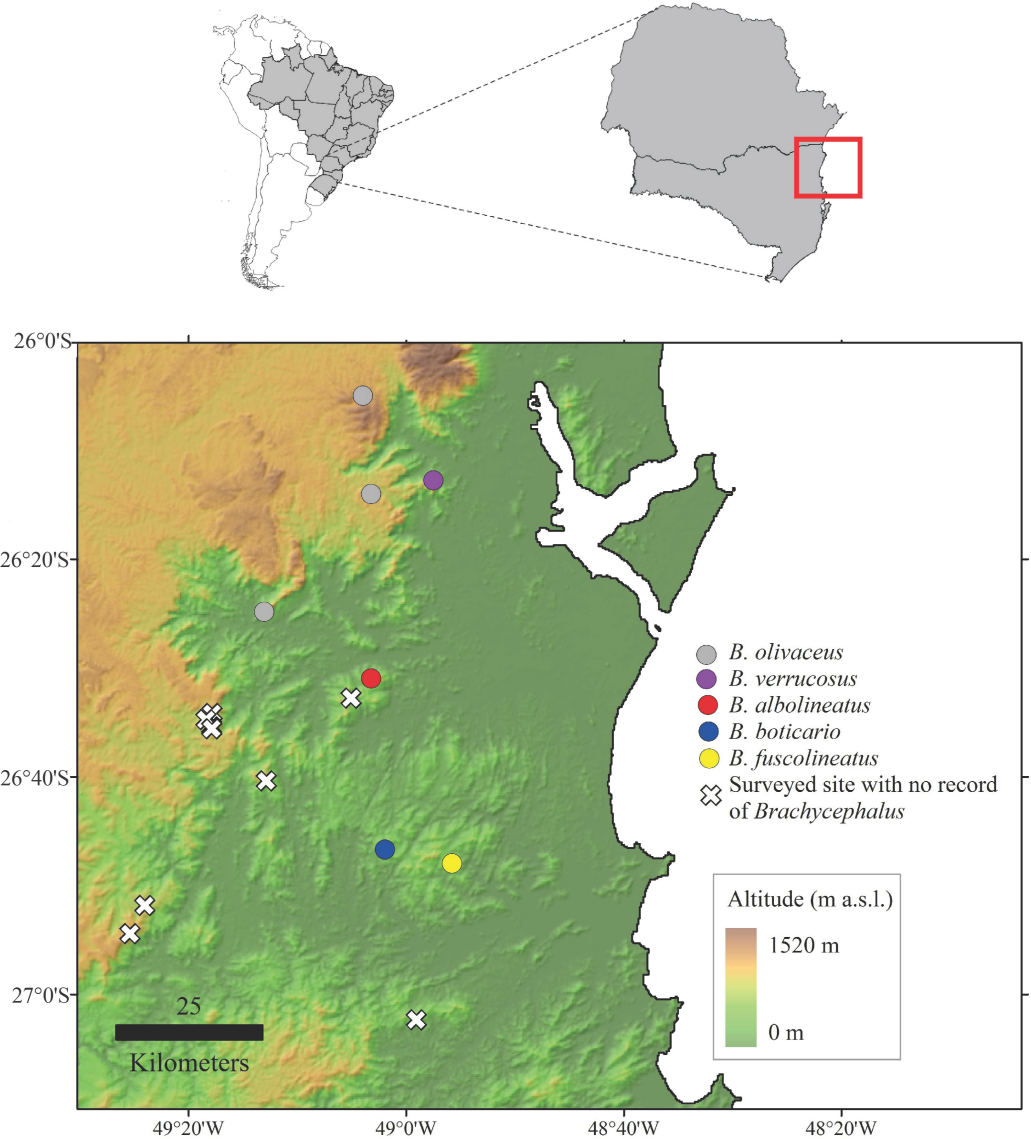
Map indicating the location of the type locality of *Brachycephalus albolineatus*(red), as well as other species of the genus that are found in the region (state of Santa Catarina, souhtern Brazil). *B. fuscolineatus* (yellow), *B. boticario* (blue), *B. olivaceus* (gray), and *B. verrucosus* (purple). White crosses indicate areas with climatic and vegetation features that are characteristic of montane *Brachycephalus* that were surveyed, but no record has been obtained to date.

**Distribution**. *Brachycephalus albolineatus* is known only from the type locality, being found in altitudes between 790–835 m a.s.l. Given the dense sampling of other potential locations with climatic and vegetation conditions similar to the type locality (Fig. 7), it likely that *B. albolineatus* has a microendemic distribution, as found in other species of the *pernix* group (Bornschein et al., 2016). For instance, we searched for the new species on a mountain named Pedra Branca just 4.8 km from the type locality (26°32′52″S, 49°05′11″W) on the border of the municipalities of Jaraguá do Sul and Massaranduba, Santa Catarina, on 6 March 2016. In this mountain, we worked from 700 m a.s.l. up to the top, at 730 m a.s.l., and we did not find the new species. This locality is also characterized by montane forest (Floresta Ombrófila Densa Montana; with canopy height between 16–22 m). We recorded a variety of species in the type locality that are typical of high altitudes, such as *Chusquea* sp., *Quelusia regia* Vell., and *Weinmannia* sp., in the case of plants, and *Attila phoenicurus* Pelzeln, 1868, *Clytolaema rubricauda* (Boddaert, 1783), *Piculus aurulentus* (Temminck, 1821), *Poecilotriccus plumbeiceps* (Lafresnaye, 1846), *Scytalopus speluncae* (Ménétriès, 1835) (taxonomy according to Maurício et al. (2010)), and *Stephanophorus diadematus* (Temminck, 1823), in the case of birds. In the other hand, we also recorded some plants in the type locality, that are typical of lowland habitats (e.g., *Bathysa australis* (A.St.-Hil.) K.Schum., *Cecropia* sp., and *Euterpe edulis* Mart.), showing a mixed flora from both high and low elevations.

**Figure 8:**
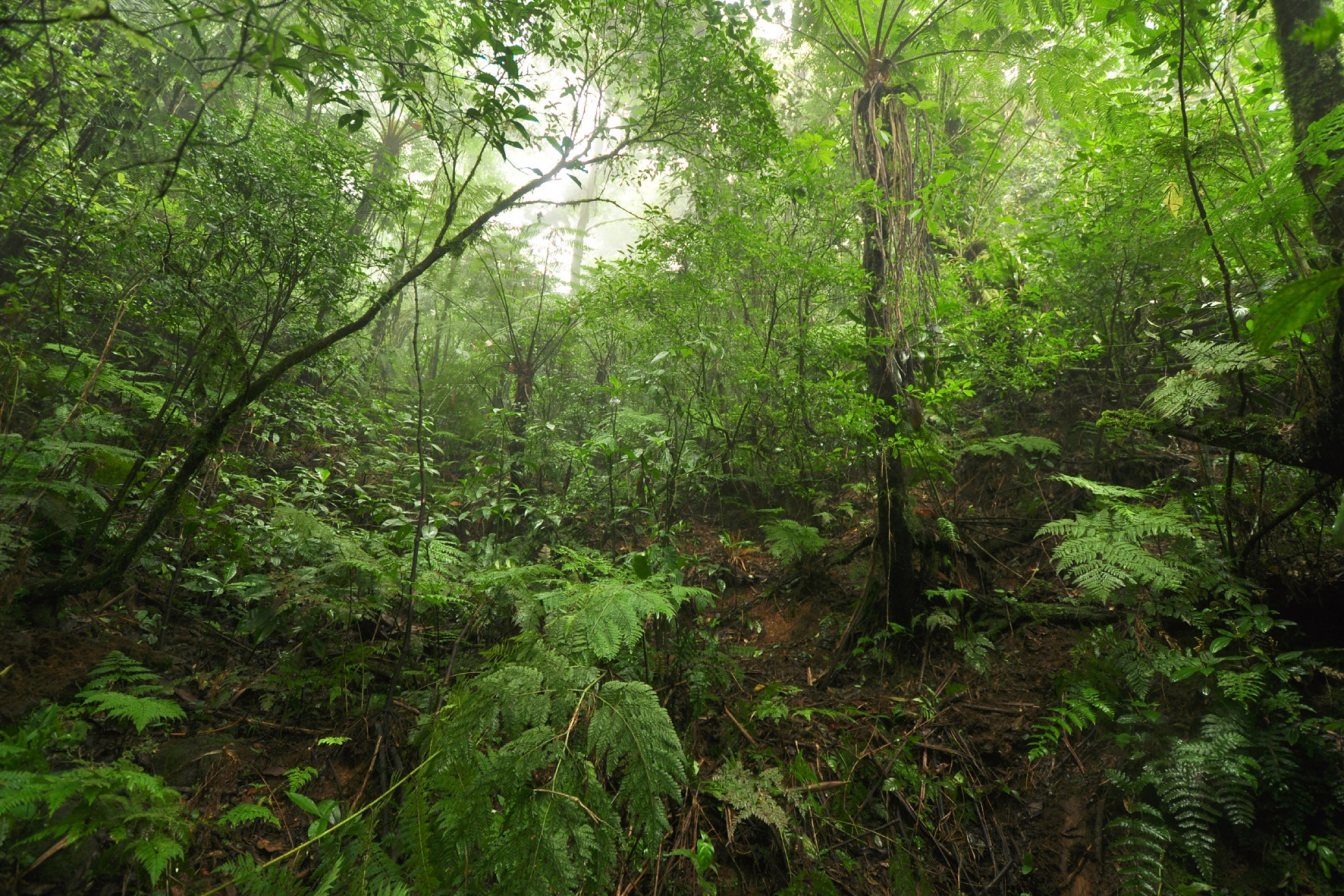
General view of the vegetation in the type locality (at 830 m above sea level).

**Ecology**. *Brachycephalus albolineatus* lives on the leaf litter of the forest floor of montane forests (Floresta Ombrófila Densa Montana; with a canopy between 10–18 m in height; Fig. 8). While we disturbed the litter while searching for specimens, some individuals were seen moving further down, with some specimens being detected in the soil between roots. It was raining on 25 October 2012 we heard no calling activity; we only collected one individual by randomly searching the leaf litter. On 5 and 6 February 2016, the species showed high calling activity on the upper limit of their occurrence (830 m a.s.l.), becoming gradually scarcer downward. We estimate about one calling individual in each 3–4 m^2^ at 815–835 m a.s.l. and every 100 m^2^ at 790 m a.s.l., respectively in the highest and lowest altitudinal limits of records of the species. On 05 and 06 February 2016, we verified that the species became silent after the sunset (at least for 30 min, when we left the site), and on 6 March 2016 we did not hear any individual calling at night (we arrived at the site 30 min after sunset and waited for 20 min).

## Discussion

Many osteological characters present in *B. albolineatus* are typical of the genus, such as the robust neurocranium, lack of teeth, and a robust operculum covering the fenestra ovalis (Da Silva, Campos & Sebben, 2007). Other traits are more typical of the *pernix* group, such as the lack of hyperossification, a slender quadratojugal, a relatively robust pterygoid, and a “complete” squamosal with a prominent zygomatic ramus. Some characters are unusual with respect to other members of this species group that have been studied to date (i.e., *B. ferruginus*, *B. pombali*, *B. brunneus*, *B. izecksohni*, and *B. pernix*; Alves et al. (2006)). This includes the apparent coossification of the sphenethmoid with the parasphenoid and the presence of the neopalatine, which is present though reduced in *B. brunneus* and *B. izecksohni* (Alves et al., 2006). Interestingly, the arytenoid cartilages of *B. albolineatus* are heavily mineralized, which has not been reported for other *Brachycephalus* species and is generally rare among frogs (Duellman & Trueb, 1986). Another interesting trait of *B. albolineatus* is the fusion of the last presacral vertebra (VIII) and the sacral vertebra, which is possibly shared with *B. brunneus* and *B. pombali* (based on Fig. 7 of Clemente-Carvalho et al. (2009)). Presacral vertebral fusions, including intraspecific variation, are well documented in *B. ephippium* but these do not include fusion of the presacral vertebrae to the sacral vertebra (Campos, Da Silva & Sebben, 2010). There is no variation in the phalangeal formula of the hand in the *Brachycephalus* species studied to date (14 species; see the list below; the manus formula of *B. didactylus*, 2-2-3-2, of Izecksohn (1988), needs confirmation). However, there is variation in the number of phalanges in toes I and V. Toe I can either have one phalanx (*B. ephippium*, *B. ferruginus*, *B. garbeanus*, *B. guarani*, *B. hermogenesi*, *B. nodoterga*, * B. pernix*, *B. pitanga*, and *B. pombali*), as in *B. albolineatus*, or lack phalanges entirely (*B. alipioi*, *B. brunneus*, *B. didactylus*, *B. ephippium*, *B. hermogenesi*, *B. izecksohni*, *B. nodoterga*, and *B. toby*). Toe V can lack phalanges (*B. brunneus*, *B. ephippium*, * B. guarani*, *B. izecksohni*, and *B. pernix*), as in the new species, have one phalanx (*B. alipioi*, *B. didactylus*, *B. ephippium*, *B. ferruginus*, *B. garbeanus*, *B. nodoterga*, *B. pitanga*, *B. pombali*, and* B. toby*), or two phalanges (*B. ephippium* and *B. hermogenesi*) (Izecksohn, 1988; Alberch & Gale, 1985; Pombal Jr, Wistuba & Bornschein, 1998; Ribeiro et al., 2005; Alves et al., 2006; Pombal Jr & Gasparini, 2006; Da Silva, Campos & Sebben, 2007; Campos, 2011; Clemente-Carvalho et al., 2012). In general, these results underscore the substantial osteological variation in *Brachycephalus*, particularly in the *pernix* group, as well as the potential for Micro-CT for providing valuable new information for osteological studies of these miniature frogs.

Bornschein et al. (2016) and Firkowski et al. (2016) postulated that altitudinal migration and population isolation during the climatic variation that occurred during the Quaternary could explain the richness and the relatively widespread distribution of montane *Brachycephalus* throughout the Atlantic Forest. In particular, during cold and dry periods, montane forests would have migrated into lower altitudes and allowed ancestral lineages to be widespread, whereas hot and wet periods led montane forests to shift upwards to mountain tops, isolating populations and leading to speciation (Bornschein et al., 2016; Firkowski et al., 2016). The discovery of *B. albolineatus* reinforces this biogeographic scenario, given that it is more one endemic montane species in an area for which the genus had not been recorded previously, allowing us to infer the extent to which montane forests shifted downward during cold and dry periods (see below). Moreover, the discovery of another montane species underscores the possibility that several additional species can be discovered in mountains with similar environmental conditions throughout the Atlantic forest, as well as the importance of these montane frogs as a model system to uncover the evolutionary processes that shaped the montane fauna that we see today, as well as to establish conservation priorities.

*Brachycephalus albolineatus*, as well as the other montane microendemics *B. boticario* and *B. fuscolineatus*, is located in an area surrounded by low-altitude rivers or valleys (Fig. 7). Apart from these species, the closest known record of *Brachycephalus* is Morro do Boi, municipality of Corupá (Bornschein et al., 2016), which is located 20 km to the northwest of *B. albolineatus*. A connection between these species would necessarily involve a shift downward to nearly 300 m a.s.l., in a passage to the southwest. On the other hand, connections between *B. albolineatus* and *B. boticario* or between *B. albolineatus* and *B. fuscolineatus* would involve shifts to even lower altitudes, down to 180 m a.s.l., whereas contact between *B boticario* and *B. fuscolineatus* would in turn involve a shift to an altitude of 275 m a.s.l., yet the lowest known record for a montane *Brachycephalus* is northeastern of Santa Catarina, at 455 m a.s.l. (Bornschein et al., 2016). The conditions involving these species is markedly different from those occurring further north in Santa Catarina and Paraná, where the lowest valleys isolating populations are often as high as 800 m a.s.l., suggesting that the climatic conditions during the cold and dry periods during the Quaternary were even more severe than previously thought.

The new species potentially occurs in an “extent of occurrence” polygon (*sensu* IUCN, 2012) of 25.04 ha, considering the lowest altitudinal limit recorded for the species as a criterion to delimit the polygon and excluding deforested areas. Only 1.08 km away from the type locality (and 522 m away from the limit of the extent of occurrence), there is an area of 9.33 ha of montane forest above 790 m a.s.l. (locally reaching up to 860 m a.s.l.). The region between these two areas involves a “valley” at 730 m a.s.l. Assuming the presence of the new species also in this second area, we will have a total area of extent of occurrence of 34.37 ha. Although we excluded open areas (e.g., deforested areas, roads, rocky areas) for polygon delimitation, even so we treated the results as extent of occurrence and not “area of occupancy” (*sensu* IUCN, 2012) because microhabitats requirements can prevent the species occurrence in some parts of the delimitated polygons (Bornschein et al., 2016). Around the type locality, there was a focus of deforestation (for roads and radio stations) of 1.46 ha, but there is no immediate expectation of expansion of the deforestation because of limitations due to its high slopes. Other types of impact, however, are potentially severe and involve invasion of exotic plants into the forest and degradation of the lower forest strata through the deposition of trash (e.g., bricks, plastic bottles, glass bottles, construction wood), which in turn can reduce the effective habitat usable (= “area of occupancy”). We also highlight the potential impacts of fires and landslides. By its highly reduced extent of occurrence (see Bornschein et al., 2016) and inferred loss of area of occupancy (at least of quality of area of occupancy), the new species matches the IUCN (2012) criteria to evaluating the conservation status of species as “critically endangered” (B1: a, b (ii, iii)). However, we propose that the species be considered as “Data Deficient” due to the possibly that new localities could still be discovered.

##  Supplemental Information

10.7717/peerj.2629/supp-1 Appendix S1 List of examined specimens

## Additional Information and Declarations

### Data Availability

The following information was supplied regarding data availability: MorphoSource: http://morphosource.org/Detail/MediaDetail/Show/media_id/10212.

### New Species Registration

The following information was supplied regarding the registration of a newly described species: *Brachycephalus albolineatus* sp nov. urn:lsid:zoobank.org:act:250B70C4-8FB0-44B6-9D05-CC2955E0748F. Publication LSID: urn:lsid:zoobank.org:pub:32F6D8BC-7B15-4A81-BC82-D4DEE1B38DD8.
