# Supplementary material for: A new species of Brachycephalus (Anura: Brachycephalidae) from Santa Catarina, southern Brazil
Source: PeerJ. 2016 Oct 27;4:e2629. doi: 10.7717/peerj.2629 (PMC5088579; doi:10.7717/peerj.2629)
Supplement: Appendix S1 [file peerj-04-2629-s001.docx]

Appendix I. Examined specimens.

*Brachycephalus auroguttatus*. SANTA CATARINA: Pedra da Tartaruga, municipality of Garuva DZUP 375 (holotype), DZUP 373-4, 376-85, 387-89 (all paratypes).

*Brachycephalus boticario*. SANTA CATARINA: Morro do Cachorro, on the border between the municipalities of Blumenau, Gaspar, and Luiz Alves DZUP 440 (holotype), DZUP 414-5, 438-9, 444-5, 459 (all paratypes).

*Brachycephalus brunneus*. PARANÁ: Caratuva, Serra dos Órgãos, municipality of Campina Grande do Sul MHNCI 1919-20, MNRJ 40289-91 (paratypes).

*Brachycephalus didactylus*. RIO DE JANEIRO: municipality of Engenheiro Paulo de Frontin ZUEC 10825, MZUSP 94621; Sacra Família do Tinguá, municipality of Engenheiro Paulo de Frontin ZUEC 1132-3, MZUSP 13613-20, 64810-1, 94621.

*Brachycephalus ephippium*. RIO DE JANEIRO: Parque Nacional Serra dos Órgãos, MZUSP 104140-7. SÃO PAULO: municipality of Cotia MHNCI 2611-16.

*Brachycephalus ferruginus*. PARANÁ: Olimpo, Serra do Marumbi, municipality of Morretes MHNCI 125, 128.

*Brachycephalus fuscolineatus*. SANTA CATARINA: Morro do Baú, municipality of Ilhota DZUP 159 (holotype), DZUP 158, 160, 401-5 (all paratypes).

*Brachycephalus hermogenesi*. SÃO PAULO: Ubatuba ZUEC 9715 (holotype), ZUEC 9716-25 (paratypes).

*Brachycephalus izecksohni*. PARANÁ: Torre da Prata, Serra da Prata, on the border between the municipalities of Morretes, Paranaguá, and Guaratuba CFBH 7381-2, 7384 (all paratypes).

*Brachycephalus leopardus*. PARANÁ: Serra do Araçatuba, municipality of Tijucas do Sul DZUP 490 (holotype), DZUP 478-89, 491-2 (all paratypes); Morro dos Perdidos, municipality of Guaratuba DZUP 274-83.

*Brachycephalus mariaeterezae*. SANTA CATARINA: Reserva Particular do Patrimônio Natural Caetezal, top of the Serra Queimada, municipality of Joinville MHNCI 9811 (holotype), DZUP 372, 393-9 (all paratypes).

*Brachycephalus nodoterga*. SÃO PAULO: Reserva Biológica Tamboré, municipality of Santana de Parnaíba MZUSP 147711-6.

*Brachycephalus olivaceus.* SANTA CATARINA: base of the Serra Queimada, municipality of Joinville MHNCI 9813 (holotype), DZUP 371 (paratype); Castelo dos Bugres, municipality of Joinville MHNCI 9814-8 (paratypes); Morro do Boi, Municipality of Corupá MHNCI 10288-9.

*Brachycephalus pernix*. PARANÁ: Anhangava, Serra da Baitaca, municipality of Quatro Barras MNRJ 17349 (holotype), CFBH 2597-8 (paratypes), MHNCI 1818-9, 3000-4 (all paratypes), MHNCI 1820, ZUEC 9433-7 (paratypes), DZUP 539-55.

*Brachycephalus pitanga*. SÃO PAULO: SP 125, municipality of São Luís do Paraitinga DZUP 407-9.

*Brachycephalus pombali.* PARANÁ: Morro dos Padres, Pico da Igreja, municipality of Guaratuba CFBH 8042 (holotype), 8043-53 (paratypes).

*Brachycephalus quiririensis*. SANTA CATARINA: Serra do Quiriri, municipality of Campo Alegre DZUP 172 (holotype), DZUP 171, 173-6, 524-30 (all paratypes).

*Brachycephalus sulfuratus*. SÃO PAULO: base of the Serra Água Limpa, municipality of Apiaí DZUP 362. PARANÁ: Caratuval, near the Parque Estadual das Lauráceas, municipality of Adrianópolis DZUP 139; Corvo, municipality of Quatro Barras DZUP 150-7; Fazenda Thalia, municipality of Balsa Nova DZUP 221-4; Mananciais da Serra, municipality of Piraquara MHNCI 10302; Recanto das Hortências, municipality of São José dos Pinhais DZUP 463; Salto do Inferno, Rio Capivari, municipality of Bocaiúva do Sul MHNCI 9800.

*Brachycephalus verrucosus*. SANTA CATARINA: Morro da Tromba, municipality of Joinville MHNCI 9819 (holotype), MHNCI 9820 (paratype), DZUP 464-78 (paratypes).

*Brachycephalus tridactylus*. PARANÁ: Serra do Morato, Reserva Natural Salto Morato, municipality of Guaraqueçaba DZUP 493-7.
